# Network Pharmacology-Based Strategy for Predicting Therapy Targets of Citri Reticulatae Pericarpium on Myocardial Hypertrophy

**DOI:** 10.1155/2022/4293265

**Published:** 2022-03-02

**Authors:** Shisheng Jiang, Chaoming Huang, Shulin Wang, Biyun Huang, Dan Wu, Guodong Zheng, Yi Cai

**Affiliations:** ^1^Key Laboratory of Molecular Target & Clinical Pharmacology and the State & NMPA Key Laboratory of Respiratory Disease, School of Pharmaceutical Sciences & The Fifth Affiliated Hospital, Guangzhou Medical University, Guangzhou 511436, China; ^2^The Sixth Affiliated Hospital of Guangzhou Medical University, Qingyuan People's Hospital, Guangzhou Medical University, Guangzhou, Qingyuan 511500, China

## Abstract

**Objective:**

Through a network pharmacology method, we screened the main active compounds of Citri Reticulatae Pericarpium (CRP), constructed a drug-ingredient-disease-target network, explored the molecular mechanism of its treatment of myocardial hypertrophy, and validated it by using molecular biology approach.

**Methods:**

Traditional Chinese Medicine Systems Pharmacology (TCMSP) and GeneCards were utilised to collect the effective component in CRP and the targets of CRP and myocardial hypertrophy. The STRING database constructed the protein interaction network. The drug-ingredient-disease-target network was outlined by the Cytoscape 3.9.0 software. The Gene Ontology (GO) and Kyoto Encyclopedia of Genes and Genomes (KEGG) pathway enrichment analyses were conducted using the Metascape database. Real-time PCR (RT-PCR) and Western blotting were utilised to determine the mRNA and protein level of the critical targets of CRP therapy for myocardial hypertrophy.

**Results:**

We found that five practical components of CRP exerted therapeutic effects on myocardial hypertrophy by modulating 41 targets. Further analysis revealed that naringenin was the essential active compound in CRP that regulated myocardial hypertrophy. In addition, we showed that the active compounds of CRP might exert antihypertrophy effects via regulating essential target proteins such as AKT1-, MAPK3-, PPARA-, PPARG-, and ESR1-mediated signaling pathways such as cell proliferation, nuclear receptor activation, and oxidative stress. The molecular biology experiments demonstrated that naringenin inhibited the mRNA level of NPPA and NPPB induced by Ang II and regulated related targets such as AKT1, MAPK3, PPARA, PPARG, and ESR1.

**Conclusion:**

CRP could inhibit myocardial hypertrophy through multitarget and multiapproach.

## 1. Introduction

Cardiovascular diseases (CVD) have become the “number one killer” threatening human health. Studies show that CVD is a primary cause of death in China, accounting for more than 40% of worldwide deaths, affecting hundreds of millions of people each year [1–3]. Myocardial hypertrophy is an everyday pathological basis and an independent risk factor for many CVD such as atherosclerosis, coronary artery disease, and valvular disease, mainly manifested by an increased surface area of cardiac myocytes, increased synthesis of cardiac proteins, and abnormal activation of embryonic genes. While early myocardial hypertrophy helps maintain average cardiac output and cardiac function, persistent pathological myocardial hypertrophy can trigger a decline in heart function and eventually lead to heart failure [4, 5]. The pathogenesis of myocardial hypertrophy is a complex multifactorial process that touches on several cellular and molecular systems. Several signaling pathways, including the CaMKII pathway, mitogen-activated protein kinase (MAPK), peroxisome proliferator-activated receptor (PPAR), PI3K/AKT, and oxidative stress response pathway, are involved in the progression of myocardial hypertrophy [6–9]. Western drugs such as angiotensin-converting enzyme inhibitors (ACEI), aldosterone receptor antagonists, and *β*-blockers are mainly used in the clinical treatment of cardiac failure, which has not significantly reduced the mortality rate [10–12]. However, they have improved the clinical symptoms of patients to some extent. Chinese medicine emphasizes the treatment of both the symptoms and the root cause of the disease and identifying evidence. More and more studies have shown that traditional Chinese medicine (TCM) is widely valued for its stable efficacy and ability to act on multiple aspects of the development of myocardial hypertrophy [13].

Citri reticulatae pericarpium (CRP), commonly called Chenpi in Chinese, is the dried and ripe peel of Citrus reticulata Blanco and its cultivated varieties, aromatic, spicy, and bitter [14]. As one of the most commonly used TCM, CRP is rich in bioactive substances, such as flavonoids and volatile, volatile compounds, oils, and polysaccharides. These active ingredients have various biological activities and medicinal values, such as antioxidant, anti-inflammatory, antibacterial, anticancer, and cardiovascular protective functions [15]. In recent years, many components in CRP, especially flavonoid glycosides, such as naringenin and hesperidin, and polymethoxyflavonoids, such as nobiletin, are played in place of CVD which have become a hot research topic [16]. Several lines of evidence have proven that these flavonoids in CRP are significant in inhibiting atherosclerosis, regulating blood lipids, and improving myocardial hypertrophy [17]. Also, a previous study demonstrated the epidemiological association between the intake of foods containing citrus flavonoids and a reduction in CVD occurrence [18]. However, to date, studies on the role and mechanism of active ingredients in myocardial hypertrophy in CRP are still incomplete and deserve further investigation.

**Figure 1:**
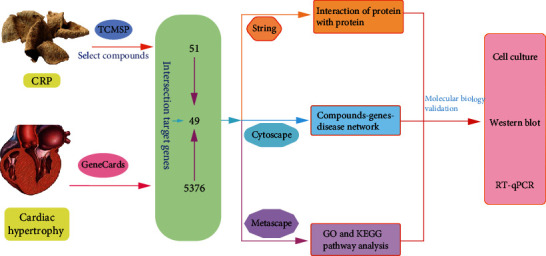
The workflow of key target gene prediction and validation of CRP therapy for myocardial hypertrophy.

This present study constructs the interaction network between the active components of CRP, drug targets, and myocardial hypertrophy-related target genes, initially investigating the molecular mechanism of action of CRP in the treatment of myocardial hypertrophy. Moreover, we also investigate the effect of naringenin on myocardial hypertrophy and related targets predicted before. The workflow is shown in Figure 1.

## 2. Materials and Methods

### 2.1. Reagents

Naringenin (purity >95%) was purchased from Aladdin Chemistry Co., Ltd. (Shanghai, China). Dulbecco's Modified Eagle Medium (DMEM) and fetal bovine serum (FBS) were purchased from Gibco (Logan, UT, USA). Real-time PCR kit was purchased from Takara Co., Ltd. (Dalian, China). Rabbit anti-ERK1/2 (#4695), anti-p-ERK1/2 (#8544), anti-AKT (#4691), and anti-p-AKT (#4060) antibodies were purchased from CST Company (Boston, MA, USA). The chemiluminescent substrate was purchased from Pierce (Rockford, IL, USA).

### 2.2. CRP Active Ingredients and Target Collection

The Traditional Chinese Medicine Systems Pharmacology Database and Analysis Platform (TCMSP) was applied to retrieve the active ingredients of CRP with the keywords of Citri reticulatae pericarpium. Oral bioavailability (OB) ≥ 30% and drug-likeness (DL) ≥ 0.18 were set as the screening criteria to screen out the active components of CRP. The active ingredients of CRP-related target genes were also obtained from the TCMSP database.

### 2.3. Disease-Target Network Construction

GeneCards (https://www.genecards.org/), an online tool, is a comprehensive resource of human genes, providing all known and predicted human-related genes in proteome, genome, genetics, transcription, and function. In this study, the GeneCards database was used to search for myocardial hypertrophy-related genes using the keyword of myocardial hypertrophy. The gene target names were corrected using Perl's computer programming language (https://www.perl.org/).

### 2.4. Clustering of CRP- and Myocardial Hypertrophy-Related Target Genes

Based on the Venn Diagram program running R statistical programming language, gene mapping was carried out on the online Venny 2.1.0 platform to find the intersection of drug targets and genes related to myocardial hypertrophy, namely drug-disease coacting target genes. And then, the target information of the active component compounds of CRP and the target information of myocardial hypertrophy were classified and stored, and a Venny diagram was drawn.

### 2.5. Protein-Protein Interaction (PPI) Network Construction and Analysis

The information of active ingredients of CRP and myocardial hypertrophy targets was imported into the network visualization software Cytoscape 3.9.0 (https://cytoscape.org/), and an optical network topology diagram of CRP-active ingredient-myocardial hypertrophy was constructed based on the Cytoscape software. The network described the relationship between the active components of CRP and myocardial hypertrophy. The active ingredients, drugs, and disease genes were nodes of the web, and the line between each node represented the relationship between the three.

### 2.6. Data Processing and Analysis

With the intersection gene of CRP-myocardial hypertrophy entered on the STRING database (https://string-db.org/), the species selected was human. Then, the protein interaction network relationships were mapped, and the data were analyzed by Cytoscape 3.9.0 to filter out the core components of the protein interaction network. Then, the intersection target genes were placed into the Metascape database, and species were selected as “Homo sapiens,” and *P* < 0.01 were set for gene ontology (GO) and Kyoto Encyclopedia of Genes and Genomes (KEGG) analysis.

### 2.7. Cell Culture

H9C2 cardiomyocytes were grown in DMEM supplemented with 10% fetal bovine serum (FBS) and maintained at 37°C in a humidified atmosphere of 95% air-5% CO_2_. The cells were digested by trypsin-EDTA (0.25%, Sigma) when they reached 80%-90% fusion and then passaged. Before treatment, the cells were treated with DMEM medium containing 0.1% FBS for 12 h to treatment to synchronize the cells, and then, subsequent experiments were performed.

### 2.8. Real-Time PCR

After H9C2 cardiomyocytes were treated with naringenin and Ang II, the culture medium was discarded, and the total RNA of cardiomyocytes was extracted with TRIzol. The mRNA concentration was detected by Bio-Rad quantitative PCR kit. PCR primers were designed using the sequences shown in Table 1, and GAPDH was utilised as an endogenous control. Three replicate wells were set up for each group of samples to ensure the validity of the experimental data.

### 2.9. Western Blotting

The extracted total cell protein was added to 2× SDS buffer, followed by SDS-PAGE gel electrophoresis, membrane transfer, blocking, the addition of primary antibody (anti-AKT, anti-p-AKT, anti-ERK1/2, and anti-p-ERK1/2), incubation at 4°C overnight, incubation at room temperature for one h on the next day with secondary antibody, detection of target protein expression by chemiluminescence, and grayscale analysis of bands by the ImageJ software.

### 2.10. Statistical Analysis

We use the SPSS 13.0 software for statistical analysis. The data of each group were presented as mean ± SD. One-way ANOVA was used for comparison between multiple groups. In all cases, differences were considered statistically significant with *P* < 0.05.

## 3. Results

### 3.1. CRP Active Ingredient Database Establishment

**Figure 2:**
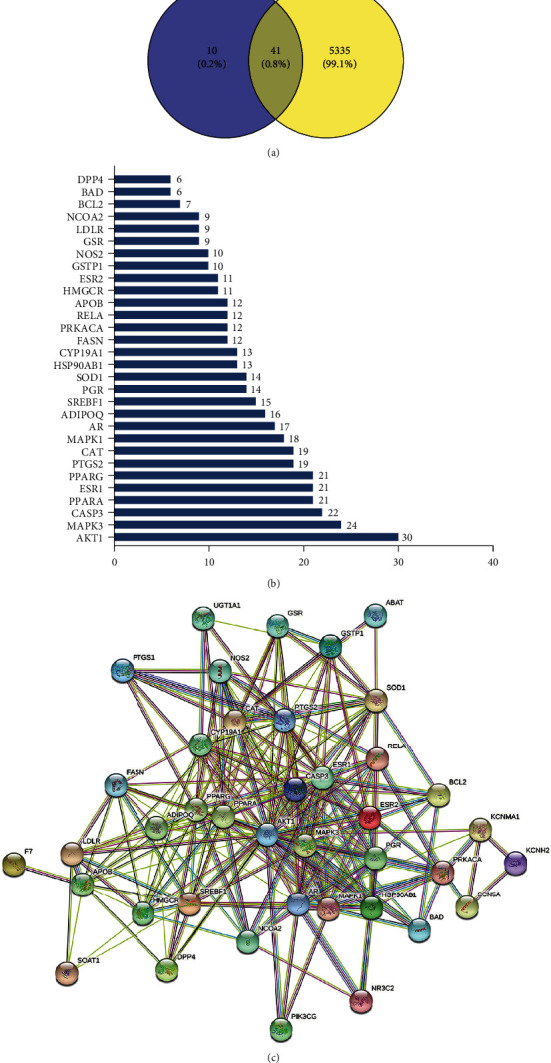
Potential target genes and PPI network map of CRP therapy for myocardial hypertrophy. (a) The Venny results of potential target genes of CRP therapy for myocardial hypertrophy. (b) The PPI network map of 41 target genes. (c) Count and list the top 30 genes of the PPI network map.

Based on the TCMSP search results, 63 active ingredients of CRP were collected. Each component's chemical information was standardized by molecular ID, molecular name, molecular weight, OB value, and DL value to establish the chemical composition information database of the drug. Subsequently, with OB ≥ 30% and DL ≥ 0.18 as criteria, five compounds with high activity were obtained by further screening, as shown in Table 2. Based on TCMSP, the active ingredient targets of CRP were obtained, the computer programming language Perl was used for name correction, and 51 marks of CRP action were obtained. The GeneCards database was used to query 5376 targets of myocardial hypertrophy disease. Based on the Venn Diagram program running R language, a total of 41 intersecting genes of CRP and myocardial hypertrophy were analyzed (Table 3 and Figure 2(a)).

OB: oral bioavailability; DL: drug-likeness.

### 3.2. Analysis of Protein-Protein Interaction Network

With the intersection targets of CRP and myocardial hypertrophy being imported into the STRING database, the free nodes outside the network being hidden, and the self-defined confidence score value > 0.4, the protein-protein interaction network of CRP-myocardial hypertrophy was carried out (Figure 2(b)). The whole network contained 233 edges, 40 nodes, and an average node degree value of 11.6. The nodes in the network represent the targets, and the edges represent the interaction between the marks. The nodes with more edges indicate that they are more critical in the network. The interaction between the nodes is supported by relevant literature evidence, with black edges representing coexpression, yellow edges representing evidence from text mining, and light blue edges representing protein homology, orange for gene fusion, etc. The cytoHubba plug-in in Cytoscape3.9.0 is used to analyze the data, calculate the nodes in the network, and draw the information histogram (Figure 2(c)). The results showed that the node degree values of target proteins such as AKT1, MAPK3, CASP3, PPARA, ESR1, and PPARG were high, indicating that these targets were in a critical position in the protein interaction network.

### 3.3. Construction and Analysis of the Drug-Component-Disease-Target Network

**Figure 3:**
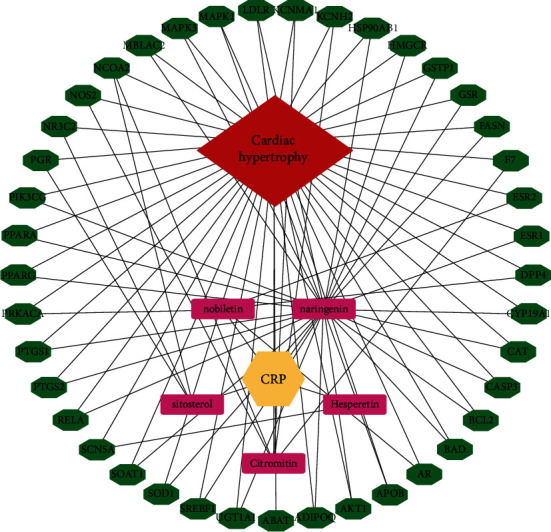
The CRP-myocardial hypertrophy-potential target gene network.

The intersections of active ingredient targets of CRP and myocardial hypertrophy disease targets were placed into Cytoscape 3.9.0, and the network of CRP-active ingredient-disease-intersection targets was mapped to elucidate the connection between the four targets (Figure 3). A total of 42 interrelationships between active ingredients of CRP and myocardial hypertrophy targets were obtained, and the critical components of CRP to inhibit myocardial hypertrophy were selected according to the parameters of Betweenness Centrality (BC), Closeness Centrality (CC), and Degree Centrality (DC). The results showed that the nodal degree value of naringenin was much higher than that of the other four compounds and was an important node in this network, suggesting that the naringenin in CRP might be a key component in the inhibition of myocardial hypertrophy (Table 4).

### 3.4. Enrichment Analysis of Biological Process and KEGG Pathway

**Figure 4:**
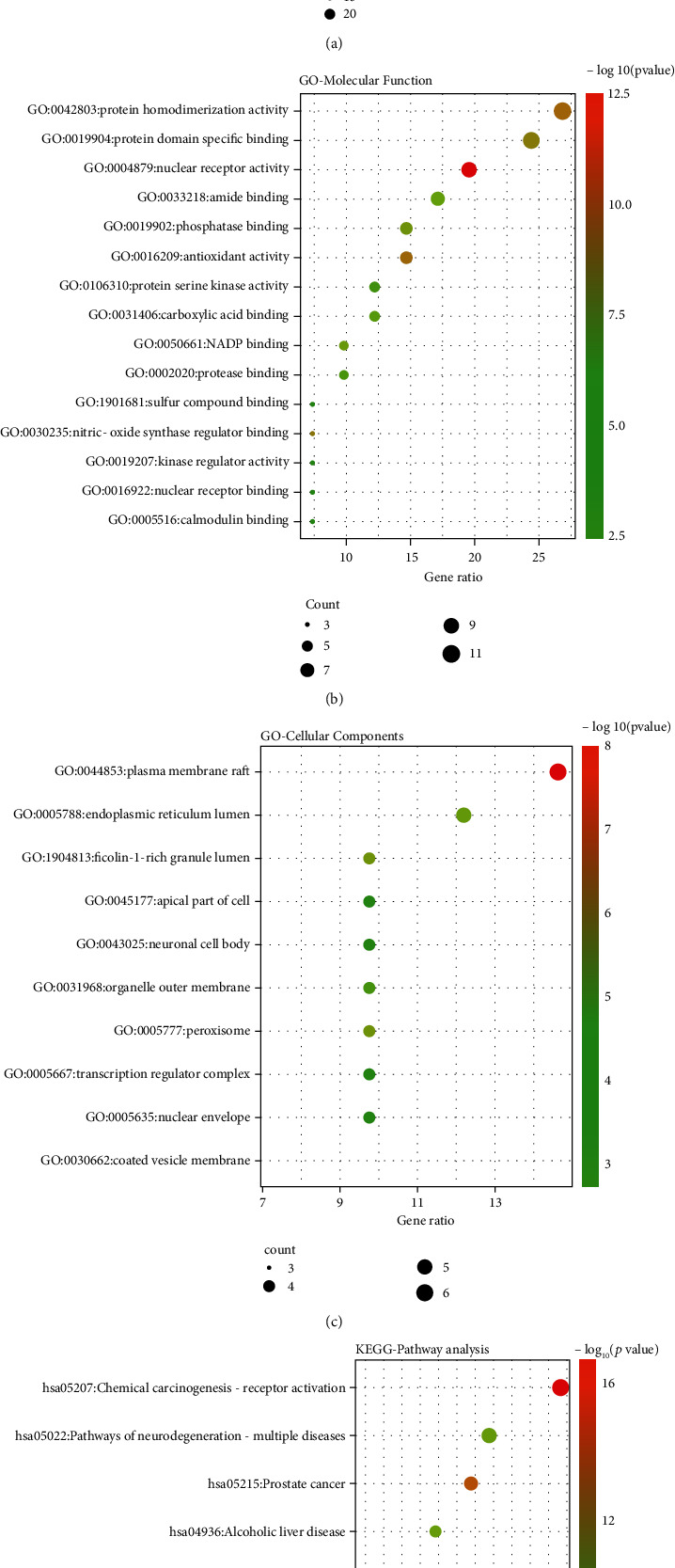
GO and KEGG analyses of potential target genes of CRP in myocardial hypertrophy. The GO analysis for biological process (a), molecular function (b), and cellular components (c) of potential target genes of CRP in myocardial hypertrophy. (d) The top 9 remarkably enriched KEGG analysis for the signaling pathway of potential target genes of CRP in myocardial hypertrophy.

Based on the annotated database of biological information, Metascape, GO bioprocess enrichment analysis, and KEGG pathway analysis of CRP-myocardial hypertrophy disease were established. The results of GO analysis obtained 20 biological processes (*P* < 0.05), 15 molecular functions (MF), and 10 cell composition (CC) corresponding to the target of the practical components of CRP for treating myocardial hypertrophy (Figures 4(a)–4(c)). Hormone (target number 22), decreased oxygen content (target number 14), oxidative stress (target number 14), and nutrient level (target number 14) were significantly enriched in the treatment of myocardial hypertrophy by CRP, suggesting that CRP could treat myocardial hypertrophy by regulating multiple complex biological processes. 138 pathways were obtained through KEGG pathway enrichment analysis (*P* < 0.01), and the top 9 pathways with higher enrichment were screened (Figure 4(d)). The results showed that the practical components of CRP could treat myocardial hypertrophy through multiple signaling pathways such as tumor-related signaling pathway (target number 16), receptor activation signaling pathway (target number 14), and nerve regeneration signaling pathway (target number 10).

### 3.5. Effects of Naringenin on Myocardial Hypertrophy Induced by Ang II

**Figure 5:**
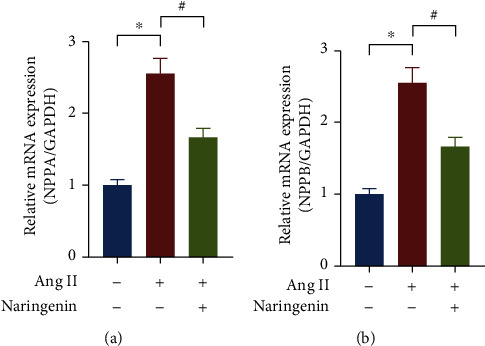
Effect of naringenin on the mRNA expression of NPPA and NPPB. H9C2 cells were treated with 20 *μ*M naringenin for 1 h followed by stimulation with Ang II for 24 h. The mRNA expressions of NPPA (a) and NPPB (b) were detected by real-time PCR. ^∗^*P* < 0.05 vs. the group without treatment, ^#^*P* < 0.05 vs. the group treated with Ang II, *n* = 5.

To clarify the effect of naringenin on cardiomyocyte hypertrophy, H9C2 cells were pretreated with naringenin for 1 h and then treated with Ang II for 24 h. The mRNA expressions of hypertrophy-related genes NPPA and NPPB were detected. Ang II was able to induce increased mRNA level of NPPA and NPPB, while naringenin pretreatment inhibited the mRNA expression of NPPA and NPPB (Figures 5(a) and 5(b)), which suggested that naringenin could inhibit Ang II-induced cardiomyocyte hypertrophy in vitro.

### 3.6. Effects of Naringenin on the Critical Targets of CRP Therapy for Myocardial Hypertrophy

**Figure 6:**
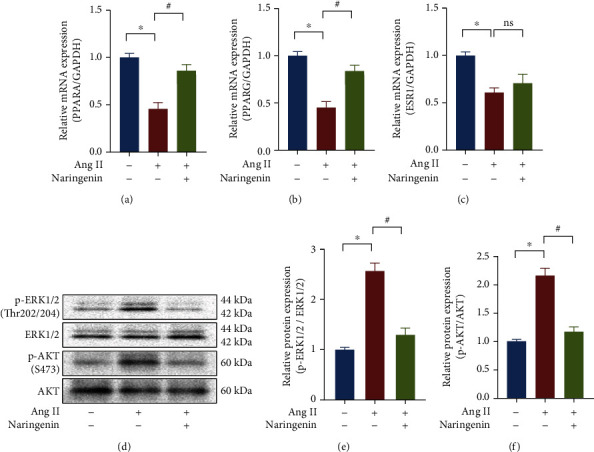
Effect of naringenin on the expression of essential target genes (AKT, PPARA, PPARG, ESR1, and ERK1/2). H9C2 cells were treated with 20 *μ*M naringenin for 1 h followed by stimulation with Ang II for 24 h. The mRNA expression of PPARA (a), PPARG (b), and ESR1 (c) were detected by real-time PCR. (d–f) The protein expression of ERK and AKT were checked by Western blotting. ^∗^*P* < 0.05 vs. the group without treatment, ^#^*P* < 0.05 vs. the group treated with Ang II, *n* = 5.

To clarify the effect of naringenin on the screened cardiac hypertrophic targets, we gave H9C2 cardiomyocytes naringenin pretreatment for 1 h, followed by Ang II treatment for 24 h. Real-time PCR detected the mRNA expression of PPARA, PPARG, and ESR1. The phosphorylation levels of AKT and ERK1/2 were detected by Western blotting. The results showed that naringenin pretreatment could inhibit the Ang II-induced decrease in mRNA expression of PPARA, PPARG, and ESR1 (Figures 6(a)–6(c)). At the same time, the administration of Ang II alone could increase the protein levels of p-AKT and p-ERK, and naringenin could inhibit the phosphorylation of AKT and ERK (Figures 6(d)–6(f)).

## 4. Discussion

Cyberpharmacology provides new ideas for Chinese medicine research by searching databases such as proteomics, genomics, and bioinformatics to perform a systematic analysis of Chinese medicine at the molecular and holistic levels to obtain the core chemical components and protein targets of TCM and to clarify the mechanism of action of TCM [19, 20]. As a traditional Chinese medicine, the role and mechanism of CRP and its various active ingredients in myocardial hypertrophy have been reported more frequently. It was found that CRP could inhibit Ang II-induced myocardial hypertrophy in mice through upregulation of PPARG [21]. Important active constituents of CRP, such as nobiletin, hesperidin, and naringenin, were also able to inhibit the development of myocardial hypertrophy. It was shown that nobiletin ameliorated pressure overload-induced myocardial hypertrophy by inhibiting oxidative stress-related signaling pathways [22]. In contrast, hesperidin may exert antimyocardial hypertrophy effects through anti-inflammatory and antioxidant pathways [8]. In addition, naringenin was also reported to inhibit diabetes-induced myocardial hypertrophy through modulation of PPAR-related pathways [23]. The above results suggested that CRP-related active components are potential candidate compounds for preventing and treating cardiovascular diseases. In this study, we collected five potent compounds of CRP, 51 targets, and 5376 disease targets of myocardial hypertrophy for gene mapping and obtained 41 intersecting genes. The critical compound naringenin and five key targets: AKT1, MAPK3, PPARA, PPARG, and ESR1, which affect myocardial hypertrophy in CRP, were obtained by analyzing the protein-protein interaction network and combining the enrichment analysis results. GO functional analysis showed that the main targets of the active ingredients of CRP in regulating myocardial hypertrophy were focused on hormone-related receptor genes, nutrition-related genes, and oxidative stress-related genes. KEGG signaling pathway enrichment analysis revealed that the active ingredients of CRP inhibited myocardial hypertrophy through tumor-related signaling pathways, receptor-activated signaling pathways, and neurodegenerative signaling pathways.

Numerous extracellular and intracellular signals synergistically regulate the onset and progression of myocardial hypertrophy [24]. Myocardial hypertrophy occurs due to an imbalance between pro and antihypertrophy factors. Several cells' signaling nodes are continuously activated during myocardial hypertrophy, and PI3K/AKT and MAPK-dependent signaling are two critical signaling pathways in the progression of myocardial hypertrophy [7, 9]. Studies have shown that various pathological stimuli, such as infarction, hypertension, and neuroendocrine factors, can activate the PI3K/AKT signaling pathway, inducing myocardial hypertrophy development. After PI3K signaling is activated, it can phosphorylate and activate Akt [25, 26]. AKT protein further regulates the transcriptional activity of myocardial hypertrophy-related transcription factors through GSK-3*β* and mTOR to initiate the expression of myocardial hypertrophy marker genes such as NPPA and NPPB [7, 27]. MAPK family, a group of serine-threonine protein kinases, plays an important role in cell proliferation, transformation, development, and inflammation [9]. MAPK-dependent signaling pathway is widely present in various cells and is involved in multiple physiopathological processes such as cell growth, proliferation, oxidative stress, inflammation, drug resistance, and autophagy [28]. The MAPK subfamily includes extracellular signal-regulated kinases (ERKs), c-Jun amino-terminal kinase (JNK), and p38 mitogen-activated protein kinase (p38-MAPK) [29]. In cardiomyocytes, in response to continuous mechanical or chemical stimulation, transforming growth factor-beta activated kinase 1 and apoptosis signal-regulating kinase 1 are activated and mediate the downstream MAPK signaling pathways ERKs, JNK, and p38-MAPK phosphorylation and shift from the cytoplasm to the nucleus [30]. ASK1 activation triggers the downstream MAPK signaling pathways ERKs, JNK, and p38-MAPK phosphorylation, which further regulates the development of myocardial hypertrophy [31]. ERK1 (MAPK3) was the first MAPK identified in mammals [32]. Previous studies have shown that G protein-coupled receptors were activated upon stimulation by extracellular hypertrophic signals, which triggered the RAS-RAF-MEK-ERK1/2 cascade signaling system, causing ERK1/2 to undergo phosphorylation translocation into the nucleus, thereby increasing the expression of hypertrophy-associated transcription factors [33]. In this present study, we predicted that AKT1 and MAPK3 might be the key targets of the active compound naringenin in Chenopodium during the prescreening process to regulate myocardial hypertrophy. Therefore, we detected the effect of naringenin on AKT and ERK phosphorylation levels in myocardial hypertrophy induced by Ang II. We showed naringenin could significantly inhibit the phosphorylation levels of AKT and ERK.

As one of the high energy-consuming tissues in the body, cardiac mitochondrial energy metabolism dysfunction is closely associated with many CVD [34]. The PPAR is highly expressed in myocardial tissues with increased mitochondrial fatty acid oxidation rates, is closely related to the homeostasis of myocardial mitochondrial energy metabolism, and is involved in processes such as cardiomyocyte differentiation and development [35]. Studies have shown that PPARA and PPARG are closely associated with the development of myocardial hypertrophy. The expression of PPARA and PPARG significantly downregulated in myocardial hypertrophy, and activation of either PPARA or PPARG was able to inhibit the hypertrophic response [35, 36]. Through some molecular experiments, we also found that naringenin significantly inhibited the Ang II-induced decrease in the expression of PPARA and PPARG, which is consistent with our predicted results.

In summary, we found that naringenin may be the critical active component in CRP that regulates myocardial hypertrophy. Moreover, we also showed that naringenin could exert inhibitory effects on myocardial hypertrophy through AKT1, MAPK3, PPARA, PPARG, and other essential target proteins mediating cell proliferation, receptor activation, oxidative stress, and different signaling pathways. Further molecular biology experiments also verified this prediction. The present study provides a scientific basis for further research on the mechanism of action of CRP against myocardial hypertrophy.

## Figures and Tables

**Table 1:** Primer sequences for qRT-PCR.

Primer	Sequences
NPPA	Forward:5′-GGAAGTCAACCCGTCTCA-3′
	Reverse:5′-AGCCCTCAGTTTGCTTTT-3′

NPPB	Forward:5′-TTTGGGCAGAAGATAGACCG-3′
	Reverse:5′-AGAAGAGCCGCAGGCAGAG-3′

PPARA	Forward:5′-TGAAAGATTCGGAAACTGC-3′
	Reverse:5′-TTCCTGCGAGTATGACCC-3′

PPARG	Forward:5′-TACCACGGTTGATTTCTC-3′
	Reverse:5′-TCTACTTTGATCGCACTTT-3′

ESR1	Forward: 5′ AGACTCGCTACTGTGCTGTG 3′
	Reverse:5′-CCTGGCAACTCTTCCTCC-3′

GAPDH	Forward:5′-AGGAGTAAGAAACCCTGGAC-3′
	Reverse:5′-CTGGGATGGAATTGTGAG-3′

**Table 2:** Characteristics of active ingredients in CRP.

No.	Molecule ID	Molecule name	Molecular weight	OB (%)	DL
1	MOL000359	Sitosterol	414.79	36.91	0.75
2	MOL004328	Naringenin	272.27	59.29	0.21
3	MOL005100	Hesperetin	302.3	47.74	0.27
4	MOL005815	Citromitin	404.45	86.9	0.51
5	MOL005828	Nobiletin	402.43	61.67	0.52

**Table 3:** 41 potential target genes of CRP therapy for myocardial hypertrophy.

No.	Target	Symbol	Entrez ID
1	Progesterone receptor	PGR	5241
2	Nuclear receptor coactivator 2	NCOA2	10499
3	Nuclear receptor subfamily 3 group C member 2	NR3C2	4306
4	Prostaglandin-endoperoxide synthase 1	PTGS1	5742
5	Estrogen receptor 1	ESR1	2099
6	Prostaglandin-endoperoxide synthase 2	PTGS2	5743
7	Heat shock protein 90 alpha family class B member 1	HSP90AB1	3326
8	Metallo-beta-lactamase domain-containing 2	MBLAC2	153364
9	Protein kinase CAMP-activated catalytic subunit alpha	PRKACA	5566
10	Phosphatidylinositol-4,5-bisphosphate 3-kinase catalytic subunit gamma	PIK3CG	5294
11	RELA proto-oncogene, NF-KB subunit	RELA	5970
12	AKT serine/threonine kinase 1	AKT1	207
13	BCL2 apoptosis regulator	BCL2	596
14	Mitogen-activated protein kinase 3	MAPK3	5595
15	Mitogen-activated protein kinase 1	MAPK1	5594
16	Caspase 3	CASP3	836
17	Fatty acid synthase	FASN	2194
18	Low-density lipoprotein receptor	LDLR	3949
19	BCL2-associated agonist of cell death	BAD	572
20	Superoxide dismutase 1	SOD1	6647
21	Catalase	CAT	847
22	Peroxisome proliferator-activated receptor gamma	PPARG	5468
23	Apolipoprotein B	APOB	338
24	3-Hydroxy-3-methylglutaryl-CoA reductase	HMGCR	3156
25	Cytochrome P450 family 19 subfamily A member 1	CYP19A1	1588
26	Glutathione S-transferase pi 1	GSTP1	2950
27	UDP glucuronosyltransferase family 1 member A1	UGT1A1	54658
28	Peroxisome proliferator-activated receptor alpha	PPARA	5465
29	Sterol regulatory element-binding transcription factor 1	SREBF1	6720
30	Glutathione-disulfide reductase	GSR	2936
31	Adiponectin, C1Q and collagen domain containing	ADIPOQ	9370
32	4-Aminobutyrate aminotransferase	ABAT	18
33	Sterol O-acyltransferase 1	SOAT1	6646
34	Sodium voltage-gated Channel alpha subunit 5	SCN5A	6331
35	Potassium voltage-gated Channel subfamily H member 2	KCNH2	3757
36	Coagulation factor VII	F7	2155
37	Potassium calcium-activated channel subfamily M alpha 1	KCNMA1	3778
38	Nitric oxide synthase 2	NOS2	4843
39	Androgen receptor	AR	367
40	Estrogen receptor 2	ESR2	2100
41	Dipeptidyl peptidase 4	DPP4	1803

**Table 4:** The list of key active components in CRP dependent on the centrality of a node.

No	Molecule name	Degree	Closeness unDir	Betweenness unDir
1	Naringenin	30	0.013157895	534.495935
2	Citromitin	6	0.008064516	30.08565434
3	Nobiletin	4	0.0078125	14.99268293
4	Sitosterol	4	0.0078125	13.7000562
5	Hesperetin	2	0.007575758	3.497560976

## Data Availability

All the data used to support the findings of this study are included in the paper.
